# Aging mothers, failing placentas? Association between maternal age and placental perfusion in a low-resource setting

**DOI:** 10.1371/journal.pgph.0005943

**Published:** 2026-02-19

**Authors:** Innocent Okafor Eze, Uchenna Ifeanyi Nwagha, Stephen Chijioke Eze, Asma’u Eleojo Abdul, Victor Cletus Igboezue, Rufai Muhammed

**Affiliations:** 1 Department of Obstetrics and Gynaecology, Nigerian Navy Reference Hospital Ojo, Lagos Nigeria; 2 Department of Clinical Sciences, School of Postgraduate Studies, University of Calabar, Cross River Nigeria; 3 Department of Obstetrics and Gynaecology, University of Nigeria Teaching Hospital, Enugu Nigeria; 4 Department of Obstetrics and Gynaecology, Federal Medical Centre Abuja, Abuja, Nigeria; 5 Department of Obstetrics and Gynaecology, Federal teaching Hospital Katsina, Katsina, Nigeria; 6 Department of Radiology, Nigerian Navy Reference Hospital, Ojo Lagos, Nigeria; Aga Khan University, PAKISTAN

## Abstract

The prevalence of pregnancies among women of advanced maternal age (AMA, ≥ 35 years) is increasing globally. Age-related vascular changes may influence placental perfusion, but evidence isolating maternal age from major confounders remains limited, particularly in low-resource settings. The objective of the study was to examine the association between maternal age and placental vascular resistance, measured using the umbilical artery pulsatility index (UA-PI). This prospective cross-sectional comparative study was conducted at a tertiary military hospital in Lagos, Nigeria. One hundred pregnancies at term were stratified into younger maternal age (<35 years, n = 50) and AMA (≥35 years, n = 50) groups. Umbilical artery Doppler assessments were performed at 37 weeks’ gestation. Mean UA-PI values were compared across maternal age groups using one-way analysis of variance with Tukey post hoc testing. Effect sizes are presented with 95% confidence intervals. At 37 weeks’ gestation, mean UA-PI was higher among women with AMA compared with younger women (0.98 ± 0.20 vs 0.80 ± 0.19; mean difference 0.18, 95% CI 0.08–0.28; p = 0.001). A progressive increase in UA-PI was observed across age categories (<25, 25–34, 35–40, ≥ 41 years; p = 0.006). Advanced maternal age was associated with higher umbilical artery vascular resistance in this cohort. These findings support further evaluation of Doppler-based placental surveillance strategies for AMA pregnancies, particularly in low-resource settings.

## Introduction

The global demographic landscape of childbirth is shifting, with an increasing proportion of women delaying pregnancy into their late thirties and beyond due to educational attainment, career considerations, delayed marriage, and the availability of assisted reproductive technologies [1,2]. Advanced maternal age (AMA), conventionally defined as 35 years or older at delivery, has been consistently associated with adverse obstetric and perinatal outcomes, including hypertensive disorders of pregnancy, gestational diabetes mellitus, fetal growth restriction, stillbirth, and increased cesarean delivery rates [3–6].

Placental dysfunction is increasingly recognized as a central mechanism underlying many age-associated pregnancy risks. Age-related vascular remodeling, endothelial dysfunction, and impaired spiral artery transformation may compromise uteroplacental and fetoplacental circulation, even in the absence of overt maternal comorbidities [7–9]. Umbilical artery Doppler velocimetry provides a non-invasive measure of placental vascular resistance and is widely used to assess placental insufficiency and fetal compromise [10].

Most existing studies examining maternal age and placental perfusion originate from high-income settings and are frequently confounded by coexisting medical conditions or focus primarily on uterine rather than fetoplacental circulation [11–13]. Evidence from low- and middle-income countries (LMICs), where antenatal care access and baseline maternal health differ substantially, remains limited.

This study therefore aimed to examine the association between maternal age and umbilical artery pulsatility index (UA-PI) in a Nigerian cohort, while excluding major medical confounders. We hypothesized that increasing maternal age would be associated with higher placental vascular resistance.

## Methods

### Ethics statement

Ethical approval was obtained from the Nigerian Navy Reference Hospital Lagos Health Research Ethics Committee (NNRHL/HREC/0012/06/2024). Written informed consent was obtained from all participants prior to enrollment. Participant confidentiality was maintained throughout the study, and all study-related investigations were provided at no cost to participants.

### Study design

This study was a prospective cross-sectional comparative study designed to examine the association between maternal age and placental vascular resistance, measured using the umbilical artery pulsatility index (UA-PI). Participants were stratified by maternal age at delivery into younger maternal age (<35 years) and advanced maternal age (AMA, ≥ 35 years). No outcome variable was used for sampling, and participants were not selected based on pregnancy outcome; therefore, the study was not a case–control design.

### Study setting and population

The study was conducted at the maternity unit of the Department of Obstetrics and Gynaecology, Nigerian Navy Reference Hospital, Ojo, Lagos, Nigeria, between 5 July 2024 and 7 March 2025. The source population comprised all pregnant women who registered for antenatal care at the facility during the study period.

### Eligibility criteria

Eligible participants were pregnant women with accurately dated pregnancies who consented to participate. Women with multifetal gestation, pregnancies conceived through assisted reproductive technologies, known fetal anomalies, or pre-existing medical conditions that could independently affect placental perfusion (including chronic hypertension, pregestational or gestational diabetes mellitus, and HIV infection) were excluded at recruitment. Women with previous pregnancy losses were not excluded unless associated with identifiable medical disorders. Smoking was excluded; this is rare in the study population.

### Sampling and recruitment

Systematic sampling was employed. The sampling frame consisted of all eligible women attending antenatal clinic during the recruitment period. Based on clinic attendance records, approximately one in five eligible women were recruited. The first participant was selected by simple random selection, after which every fifth eligible woman was approached until the target sample size was achieved. Recruitment continued until equal numbers were obtained in the younger and AMA groups.

### Sample size determination

Sample size was estimated using G*Power version 3.1.9.4. Assuming a large standardized effect size (f = 0.40) based on prior Doppler studies comparing vascular indices across maternal age groups, a two-sided alpha of 0.05, power of 0.90, and four age categories for one-way analysis of variance (ANOVA), the minimum required sample size was 84. To account for incomplete data and to ensure balanced age strata, 100 participants were recruited.

### Data collection and measurements

Sociodemographic and obstetric data were collected using a standardized questionnaire. Socioeconomic status was classified using the revised Nigerian socioeconomic classification scheme by Ibadin et al.

Gestational age was determined using the first day of the last menstrual period and confirmed by first-trimester ultrasound when available.

Umbilical artery Doppler ultrasound examinations were performed at 37 weeks’ gestation using curvilinear abdominal transducers on Mindray Siemens Acuson S1000 and S2000 ultrasound systems. All Doppler examinations were conducted by consultant radiologists trained in obstetric Doppler imaging. Umbilical artery waveforms were obtained from a free-floating loop of the umbilical cord using color Doppler guidance. Measurements were recorded in the absence of fetal breathing movements or uterine contractions. At least three consecutive uniform waveforms were obtained, and the mean value was used for analysis. The angle of insonation was kept as close to 0° as possible and always below 30°.

### Outcomes

The primary outcome was mean umbilical artery pulsatility index (UA-PI) at 37 weeks’ gestation. Secondary outcomes included the proportion of participants with UA-PI values above the 95th percentile for gestational age, defined using established reference standards.

### Statistical analysis

Data were analyzed using SPSS version 26. Continuous variables were summarized using means and standard deviations or medians and interquartile ranges as appropriate. Categorical variables were summarized using frequencies and percentages.

Distributional assumptions for parametric analyses were assessed prior to analysis. UA-PI values were approximately normally distributed, and homogeneity of variances across maternal age groups was considered acceptable; therefore, parametric tests were applied. Mean UA-PI values across maternal age categories (<25, 25–34, 35–40, and ≥41 years) were compared using one-way ANOVA. Post hoc pairwise comparisons were performed using Tukey’s honestly significant difference test when overall significance was observed.

Comparisons between younger maternal age and AMA groups were conducted using independent-samples t-tests for continuous variables and chi-square tests for categorical variables. Effect sizes are reported with 95% confidence intervals.

Given the modest sample size and the limited number of outcome events, multivariable regression analysis was not performed, as adjusted models would have been underpowered. This limitation is explicitly acknowledged.

A p-value <0.05 was considered statistically significant.

## Results

### Participants characteristics

A total of 100 pregnant women were included, comprising 50 women aged <35 years and 50 aged ≥35 years. Mean maternal age was 27.8 ± 3.2 years in the younger group and 37.4 ± 2.1 years in the AMA group. Most participants belonged to the middle socioeconomic class.

Mean maternal weight at 37 weeks’ gestation was higher in the AMA group than in the younger group but this was not statistically significant. Pre-pregnancy body mass index data were unavailable. This is shown in (Table 1).

**Table 1:** Demographic characteristics of study participants by maternal age group.

Variable	<35 years, n (%)	≥35 years, n (%)	Total, n (%)	p-value
Education				0.001
Primary	2 (100.0)	0 (0.0)	2 (2.0)	
Secondary	20 (74.1)	7 (25.9)	27 (27.0)	
Tertiary	28 (39.4)	43 (60.6)	71 (71.0)	
Ethnicity				0.031
Igbo	26 (48.1)	28 (51.9)	54 (54.0)	
Hausa/Fulani	9 (81.8)	2 (18.2)	11 (11.0)	
Yoruba	4 (36.4)	7 (63.6)	11 (11.0)	
Others	11 (45.8)	13 (54.2)	24 (24.0)	
Religion				0.050
Christianity	38 (49.4)	39 (50.6)	77 (77.0)	
Islam	10 (47.6)	11 (52.4)	21 (21.0)	
Others	2 (100.0)	0 (0.0)	2 (2.0)	
Social class				0.345
Middle	46 (53.5)	40 (46.5)	86 (86.0)	
Lower	4 (28.6)	10 (71.4)	14 (14.0)	
Parity				0.434
Nulliparous (0)	16 (66.7)	8 (33.3)	24 (24.0)	
Parity 1	21 (46.7)	24 (53.3)	45 (45.0)	
Parity ≥2	13 (41.9)	18 (58.1)	31 (31.0)	
Weight at 37 weeks				0.125
< 90 kg	32 (69.6)	14 (30.4)	46 (46.0)	
≥ 90 kg	18 (33.3)	36 (66.7)	54 (54.0)	

Values are presented as number (percentage). P-values were derived using chi-square tests. Statistical significance was defined as p < 0.05.

### Umbilical artery pulsatility index

**Fig 1:**
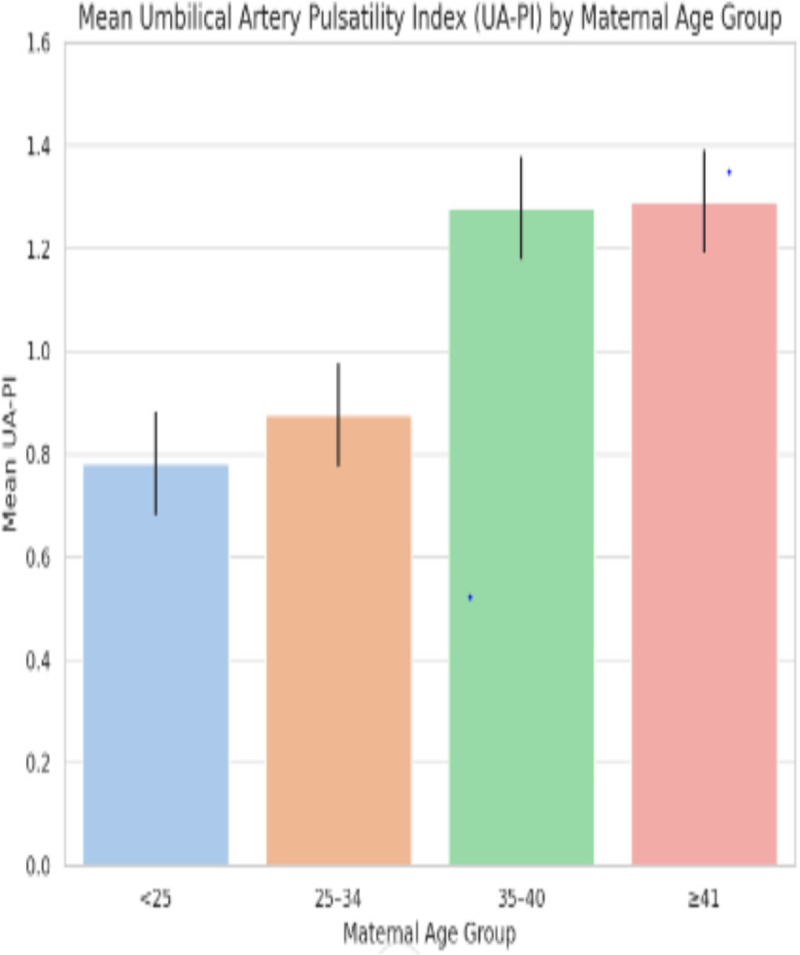
Umbilical artery pulsatility index (UA-PI) across maternal age categories. Mean UA-PI increased progressively across the maternal age groups (<25, 25–34, 35–40, ≥ 41 years). Values represent mean UA-PI for each maternal age category.

Across maternal age categories, mean UA-PI increased progressively (<25: 0.78; 25–34: 0.88; 35–40: 1.28; ≥ 41: 1.29; p = 0.006). This is shown in Fig 1.

The proportion of participants with UA-PI values above the 95th percentile was greater in the AMA group (26% vs 6%; p = 0.007) as shown in Table 2.

**Table 2:** Umbilical artery pulsatility index (UA-PI) comparison between maternal age groups.

Variable	AMA group (≥35 years, n = 50)	Younger group (20–34 years, n = 50)	p-value
Maternal age (years), mean ± SD	37.4 ± 2.1	27.8 ± 3.2	<0.001
Gestational age (weeks), mean ± SD	37.2 ± 0.5	37.3 ± 0.4	0.228
Mean UA-PI ± SD	0.98 ± 0.20	0.80 ± 0.19	0.001
UA-PI > 95th percentile, n (%)	13 (26)	3 (6)	0.007

UA-PI indicates umbilical artery pulsatility index. Continuous variables were compared using independent-samples t-tests and categorical variables using chi-square tests.

**Fig 2:**
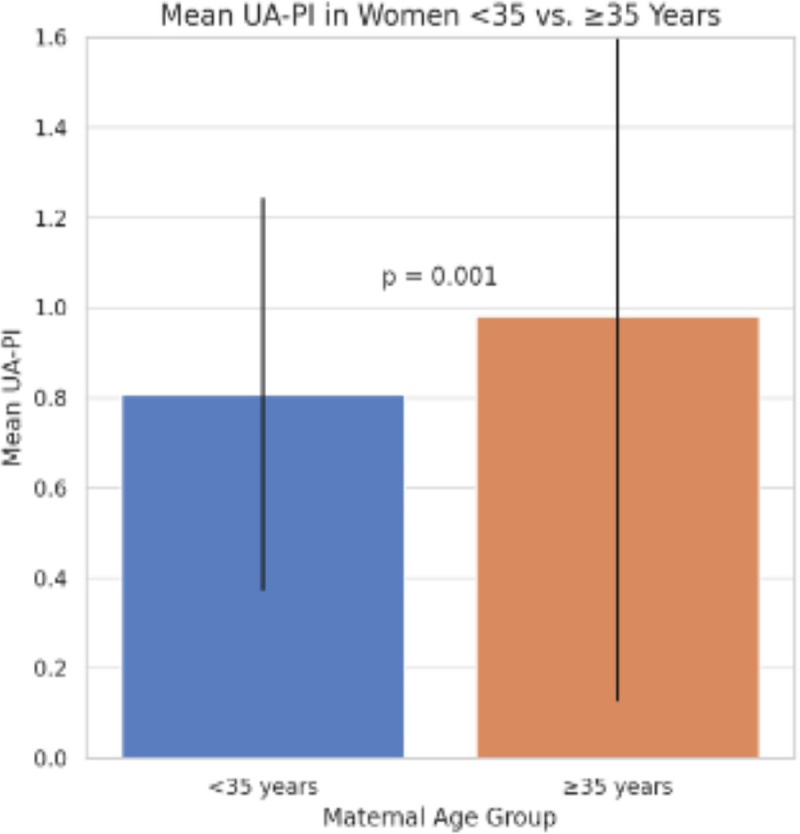
Mean umbilical artery pulsatility index (UA-PI) among younger and advanced maternal age groups at 37 weeks’ gestation. UA-PI values were significantly higher in the advanced maternal age group.

At 37 weeks’ gestation, mean UA-PI was significantly higher among women with AMA compared with younger women (0.98 ± 0.20 vs 0.80 ± 0.19; p = 0.001) as shown in Fig 2.

Post hoc analysis demonstrated significant differences between the 35–40 year group and both the < 25 and 25–34 year groups as shown in Table 3

**Table 3:** Umbilical artery pulsatility index (UA-PI) across maternal age categories.

Maternal age category (years)	n	Mean UA-PI
<25	22	0.78
25–34	28	0.88
35–40	41	1.28
≥41	9	1.29
**p-value (ANOVA)**		**0.006**

Mean UA-PI values were compared across maternal age categories using one-way analysis of variance. Post hoc pairwise comparisons were performed using Tukey’s honestly significant difference test.

## Discussion

In this prospective cross-sectional study, advanced maternal age was associated with higher umbilical artery pulsatility index, suggesting increased placental vascular resistance among older pregnant women. The graded increase in UA-PI across maternal age categories supports the biological plausibility of age-related impairment in placental perfusion.

These findings are consistent with prior reports linking maternal age to placental morphological changes, impaired angiogenesis, and altered vascular remodeling [8,9,11]. Differences between our findings and those from some high-income cohorts may reflect contextual factors, including baseline maternal health, nutritional status, and access to antenatal care.

The study was not designed to assess perinatal outcomes, and no cases of preeclampsia, gestational hypertension, stillbirth, or clinically diagnosed fetal growth restriction occurred during the study period. Consequently, inferences regarding downstream clinical outcomes cannot be made.

### Strengths and limitations

Key strengths include prospective data collection, standardized Doppler assessment by trained radiologists, and exclusion of major medical confounders. Limitations include the single-center design, modest sample size, lack of multivariable adjustment, absence of pre-pregnancy BMI data, and inability to perform placental histopathological examination.

## Conclusion

Advanced maternal age was associated with increased umbilical artery vascular resistance in this Nigerian cohort. While causality cannot be inferred, these findings highlight the need for larger, multi-center studies to clarify the role of Doppler-based placental assessment in risk stratification for AMA pregnancies in low-resource settings.
